# Berberine alters the gut microbiota metabolism and impairs spermatogenesis: Berberine alters gut microbiota metabolism and impairs spermatogenesis

**DOI:** 10.3724/abbs.2024174

**Published:** 2024-10-15

**Authors:** Wei Qu, Yumin Xu, Jing Yang, Hanqing Shi, Junli Wang, Xinnai Yu, Jiemin Chen, Binyi Wang, Deqing Zhuoga, Mengcheng Luo, Rong Liu

**Affiliations:** 1 Hubei Provincial Key Laboratory of Developmentally Originated Disease TaiKang Medical School (School of Basic Medical Sciences) Wuhan University Wuhan 430071 China; 2 Reproductive Medicine Center the Affiliated Hospital of Youjiang Medical University for Nationalities Baise 53300 China; 3 Institute of Livestock Research Tibet Academy of Agriculture and Animal Husbandry Science Lhasa 850000 China

**Keywords:** berberine, spermatogenesis, gut microbiota, testosterone, ornithine metabolism

## Abstract

Berberine (BBR) is used to treat diarrhea clinically. However, its reproductive toxicity is unclear. This study aims to investigate the impact of BBR on the male reproductive system. Intragastric BBR administration for 14 consecutive days results in a significant decrease in the serum testosterone concentration, epididymal sperm concentration, mating rate and fecundity of male mice. Testicular treatment with testosterone propionate (TP) partially reverses the damage caused by BBR to the male reproductive system. Mechanistically, the decrease in Muribaculaceae abundance in the gut microbiota of mice is the principal cause of the BBR-induced decrease in the sperm concentration. Both fecal microbiota transplantation (FMT) and polyethylene glycol (PEG) treatment demonstrate that Muribaculaceae is necessary for spermatogenesis. The intragastric administration of *Muribaculaceae intestinale* to BBR-treated mice restores the sperm concentration and testosterone levels. Metabolomic analysis reveals that BBR affects arginine and proline metabolism, of which ornithine level is downregulated. Combined analysis via 16S rRNA metagenomics sequencing and metabolomics shows that Muribaculaceae regulates ornithine level. The transcriptomic results of the testes indicate that the expressions of genes related to the low-density lipoprotein receptor (LDLR)-mediated testosterone synthesis pathway decrease after BBR administration. The transcriptional activity of the *Ldlr* gene in TM3 cells is increased with increased ornithine supplementation in the culture media, leading to increased testosterone synthesis. Overall, this study reveals an association between a BBR-induced decrease in Muribaculaceae abundance and defective spermatogenesis, providing a prospective therapeutic approach for addressing infertility-related decreases in serum testosterone triggered by changes in the gut microbiota composition.

## Introduction

The infertility rate in couples of reproductive age is as high as 15%, of which male infertility accounts for approximately 50% [1]. Over the past 40 years, the seminal sperm concentration of adult males has decreased by more than 50% in Western countries due to multiple factors, such as unhealthy diet and lifestyle, exposure to toxic chemicals, and the abuse of medications [2]. For example, accumulating evidence shows that traditional Chinese medicine (TCM) might cause reproductive dysfunction. Triptonide, a TCM-derived drug for the treatment of immune-related diseases, was reported to induce sperm malformation and decrease motility [3]. Gossypol, a toxic crystalline compound present in cotton seed oil, has also been shown to repress intercellular communication via gap junctions among Sertoli cells and steroidogenesis of Leydig cells in mouse testes [ 4, 5]. In addition, gossypol has been reported to disrupt spermatogenesis by inhibiting testis-specific lactate dehydrogenase C4 in mice [6]. Aristolochic acid induces testicular injury by inhibiting amino acid metabolism, glucose metabolism, β-oxidation of fatty acids and the tricarboxylic acid cycle in male mice [7]. Recently, berberine (BBR) has been used to treat diarrhea [8], inflammatory diseases [9], and colon cancer [10]. The daily oral administration of 0.9 g of BBR to patients receiving chemotherapy relieves chemotherapy-induced radiation intestinal syndrome [11]. A daily dose of 0.6 g BBR is effective in reducing the risk of recurrence of colorectal adenoma [12]. However, the effect of BBR on the male reproductive system remains to be elucidated.

The gut microbiota is involved in many important physiological processes of the host, including the regulation of immunity and maintaining metabolic homeostasis and cognitive functions [ 13, 14]. Many intestinal diseases can cause imbalances in the gut microbiota, including inflammatory bowel disease, irritable bowel syndrome, chronic constipation and osmotic diarrhea [15]. Diarrhea is often accompanied by changes in the osmotic pressure of the gut, leading to changes in the abundance of osmolality-sensitive microbiota. It has been reported that the osmotic laxative PEG changes the composition of the gut microbiota, resulting in a significant decrease in Muribaculaceae abundance [ 16, 17]. Numerous herbal ingredients have low bioavailability and have been shown to function in regulating the gut microbiota. Saponins were reported to facilitate the growth of beneficial bacteria and suppress cachexia-like symptoms in mice [18]. Rhein, one of the main components of rhubarb, increases the abundance of *Lactobacillus* and alleviates uric acid-induced chronic colitis [19]. Piperine improves Parkinson’s disease by decreasing the abundance of *E*. *faecalis* [20]. BBR is used to treat diarrhea, which may alter intestinal osmolality and thus the abundance of osmolality-sensitive microbiota.

Metabolomics is a systematic study of small metabolic molecules that is effective in elucidating the interaction between host metabolism and the intestinal microbiota [21]. Through combined analysis of the gut microbiota and metabolomics, FuFang Zhenshu TiaoZhi (FTZ) is shown to ameliorate the aging of mice by modulating arachidonic acid metabolism, sphingolipid metabolism, glycerophospholipid metabolism, taurine metabolism and hypotaurine metabolism [22]. The abusive use of the food additive synthetic emulsifier carboxymethylcellulose increases the prevalence of chronic inflammatory diseases by altering the gut microbiome and metabolome [23]. BBR has low bioavailability, and the gut microbiota is likely to be an important pathway for the effects of BBR. Therefore, we conducted combined analysis of the gut microbiome and metabolome to investigate the mechanism of BBR. Recent studies have focused on the role of the gut-testis axis in spermatogenesis. For example, chronic alcohol-induced dysbiosis of the gut microbiota impairs sperm quality in mice [24]. Dysbiosis of the gut microbiota induced by a high-fat diet causes impaired sperm production and motility [2]. Bacterial overgrowth in the small intestine also disrupts spermatogenesis [25]. Moreover, alginate oligosaccharides restore spermatogenesis disrupted by busulfan by increasing the abundance of *Bacteroidales* and *Lactobacillaceae* and decreasing the abundance of *Desulfovibrionaceae* [26].

In the present study, we reported that BBR administration impairs spermatogenesis in mice, which is mainly due to a decrease in the abundance of Muribaculaceae. Mechanistically, BBR leads to a decrease in Muribaculaceae abundance in the gut microbiota, which results in reduced ornithine level and subsequently decreases chromatin accessibility at the *Ldlr* promoter. A decrease in low-density lipoprotein receptor (LDLR) expression hinders LDLR-mediated testosterone synthesis and subsequent spermatogenesis.

## Materials and Methods

### Animals

Eight-week-old ICR male mice (Vital River, Beijing, China) were maintained under a light-dark cycle of 12:12 h at 23 ± 2°C. The mice had free access to food and water. All animal experiment protocols were approved by the Institutional Animal Care and Use Committee of Wuhan University. All animal experiments were performed in accordance with the Guidelines for Animal Experiments.

### BBR administration

BBR powder (Macklin, Shanghai, China) was dissolved in normal saline and shaken. On the basis of the clinical dose of BBR used for chemotherapy-induced radiation-induced intestinal syndrome treatment (0.9 g/day, *i* . *e*., 12.9 mg/kg), we converted the human dose to the mouse dose (120 mg/kg) on the basis of the body surface area [11]. Male ICR mice were randomly assigned to 4 groups (7 mice per group): the control group (only saline), the low-dose group (60 mg/kg BBR), the medium-dose group (120 mg/kg), and the high-dose group (240 mg/kg). Two weeks after continuous gavage, mouse testes, epididymides, feces, and serum samples were collected.

PEG gavage experiments were performed as described previously [17]. Briefly, 15% PEG 3350 (Bayer, Leverkusen, Germany) dissolved in water was administered intragastrically to the mice for 2 weeks, and the biological samples were collected as described above.

Testicular treatment experiments were performed as previously reported [27]. TP (NSHF, Ningbo, China) was administered subcutaneously at a dose of 10 mg/kg to the mice [ 27, 28]. Male mice were randomly assigned to 3 groups (6 mice per group): the control group (only saline), the BBR-treated group (240 mg/kg BBR), and the rescue group (240 mg/kg BBR + 10 mg/kg TP). After 2 weeks of treatment, epididymis and serum samples were collected from the mice.

### Mating behavior and fertility measurement

For the mating behavior assay, adult mice were injected intraperitoneally with pregnant mare serum gonadotropin (PMSG, 5 IU; NSHF, Ningbo, China) followed by human chorionic gonadotropin (hCG, 5 IU; NSHF) 48 h later. The male mice treated with different doses of BBR and the adult female mice treated with estrus synchronization were mated, and the copulatory plugs were checked the following morning. The male mice were considered to have mating behavior if there were copulatory plugs at the vaginal opening of the female mice. For fertility, BBR-treated mice were mated with female mice in normal oestrus, and the litter sizes were determined.

### Fecal microbiota transplantation (FMT) and bacteria supplementation experiments

Before FMT, the mice were pretreated with a cocktail of broad-spectrum antibiotics (Abx), including 0.2 g/L neomycin sulfate (Beyotime, Shanghai, China), 0.2 g/L metronidazole (Bidepharm, Shanghai, China), 0.2 g/L ampicillin (Solarbio, Shanghai, China) and 0.1 g/L vancomycin (Beyotime), which were added to the drinking water and administered to the mice for 2 weeks. Then, 400 mg of fresh stool was collected from the mice in the control or high-dose groups, resuspended in 4 mL of saline, vortexed, and filtered through a 70-μm cell strainer (Corning, New York, USA). Finally, 300 μL of fecal supernatant containing the fecal microbiota was intragastrically administered to the mice once every 2 days for 2 weeks.

Bacterial colonization supplementation experiments were performed as described below. A representative member of Muribaculaceae *(M*. *intestinale*, DSMZ 28989) was grown on chopped meat at 37°C anaerobically. A cocktail of *M*. *intestinale*was resuspended in saline at 3 × 10 ^9^ colony forming units (CFUs)/mL. The mice were intragastrically administered with *M*. *intestinale* cocktail (200 μL/mouse) once every 2 days for 2 weeks.

### Sperm counting

The cauda epididymis was removed from each mouse, placed in 1 mL of PBS buffer and minced, and the EP tube was subsequently placed at 37°C for 20 min to allow the sperm to be released into the PBS buffer. The sperm concentration was determined with a hemocytometer, and the sperm morphology was observed with an Axio Imager 2 microscope (Zeiss, Oberkochen, Germany).

### Histological examination

The testes and cauda epididymides were removed from the mice, fixed in 4% formalin, embedded in paraffin, and cut into 5-μm sections. The sections were then stained with the hematoxylin-eosin (H&E) staining kit (Beyotime) and observed under a light microscope (Olympus, Tokyo, Japan).

### Immunofluorescence staining

The testes were removed from the mice, fixed in 4% formalin, embedded in paraffin, and cut into 5-μm sections. The samples were then permeabilized and blocked. Primary antibodies and secondary antibodies were added to label the target protein, and DAPI was used to label the nucleus. Finally, the stained testis sections were observed under a fluorescence microscope. The fluorescence intensity of the sections was analyzed by using ImageJ 1.51j8 software. The antibodies used in this study are listed in Supplementary Table S1.

### Testosterone, follicle-stimulating hormone (FSH) and luteinizing hormone (LH) content measurements

Mouse serum was collected, and testosterone levels were detected via an ELISA kit (ABclonal, Wuhan, China) according to the manufacturer’s instructions. The lowest detection limit for the ELISA kit was 0.19 ng/mL. The FSH and LH levels were measured with an ELISA kit (Elabscience, Wuhan, China) according to the manufacturer’s instructions. The lowest detection limit for the FSH ELISA kit was 0.94 ng/mL, and the lowest detection limit for the LH ELISA kit was 0.19 ng/mL.

### 16S rRNA sequencing of the gut microbiota

Fresh feces were collected under sterile conditions, and fecal DNA was extracted using the Magnetic Soil and Stool DNA kit (TIANGEN, Beijing, China) according to the manufacturer’s instructions. The bacterial 16S rRNA gene sequence (V3-V4 region) was amplified using the specific primers 341F: 5′-CCTAYGGGRBGCASCAG-3′ and 806R: 5′-GGACTACNNGGGTATCTAAT-3′. Sequencing was conducted by Novogene Technology Company (Beijing, China). QIIME2 was used to conduct α-diversity analysis for OTUs with similarity levels greater than 97%. PCA plot was generated based on Bray-Curtis distance. The linear discriminant analysis (LDA) effect size (LEfSe) and an R package edge R were used to detect the differential taxons.

### Extraction of testis RNA and sequencing

Testes were harvested at the indicated time points, as described above. Total RNA was first extracted from the testes, and mRNA was purified using poly(T) oligo-attached magnetic beads. Fragmentation was carried out using divalent cations, and the fragments were reverse-transcribed into cDNA using random primers. Second-strand cDNA synthesis was subsequently performed using DNA polymerase I and RNase H. The remaining overhangs were converted into blunt ends, poly(A) was added, and the fragment products were ligated to the Illumina sequencing adaptor. The ligation fragments were size-selected using the AMPure XP system (Beckman, Pasadena, USA), and PCR amplification and sequencing on Illumina platforms with the PE150 strategy were performed by Novogene Technology Company. Differentially expressed genes were screened using the criteria of fold change > 1.5 and *P* value < 0.05.

### Real-time quantitative PCR (RT-qPCR)

Total RNA from testes or cultured cells was extracted using the Total RNA Isolation kit (Vazyme, Nanjing, China) and reverse-transcribed into cDNA using the HiScript II 1st Strand cDNA Synthesis Kit (Vazyme). The primer sets used for RT-qPCR were listed in Supplementary Table S2. RT-qPCR was then performed, and relative gene expression (fold change) was calculated using the 2 ^‒∆∆Ct^ method, in which *β-actin* was used as the internal control.

### Untargeted metabolomics by liquid chromatography-mass spectrometry (LC-MS/MS)

Fresh feces (100 mg) were collected and dissolved in methanol. For tests, testes (10 mg) were collected and dissolved in methanol. The supernatant obtained after centrifugation was used for LC-MS/MS analysis using the Vanquish UHPLC system (Thermo Fisher Scientific, Waltham, USA) coupled with an Orbitrap Q Exactive HF-X mass spectrometer (Thermo Fisher Scientific) in both positive and negative ion modes at Novogene Technology Company. Differentially abundant metabolites were identified by a fold change ≥ 2 and a *P* value < 0.05.

### Chromatin immunoprecipitation (ChIP) assays

TM3 cells were cross-linked, and the reaction was quenched with glycine. The nuclei were extracted, and the DNA was fragmented into 150‒300 bp fragments via sonication. The lysates were incubated with an anti-H3K4me3 antibody or IgG, and protein A/G agarose beads (GE Healthcare, Chicago, USA) were added. The beads were washed once with low-salt buffer, twice with high-salt buffer, twice with LiCl buffer, and twice with TE buffer. The washed beads were eluted, and crosslinking was reversed. After incubation with RNase A and proteinase K, the DNA was purified for RT-qPCR.

### Statistical analysis

Differences between two groups were compared by Student’s *t* test. For multiple comparisons, one-way ANOVA was performed using SPSS 26.0 software with Duncan’s and least significant difference (LSD) methods to identify differences among groups. Data are expressed as the mean ± SEM. *P* < 0.05 was considered to indicate statistical significance. All Kyoto Encyclopedia of Genes and Genomes (KEGG) and Gene Ontology (GO) analyses were performed on the website ( http://www.bioinformatics.com.cn).

## Results

### BBR administration impairs spermatogenesis and decreases the mating rate and fecundity in male mice

**Figure 1:**
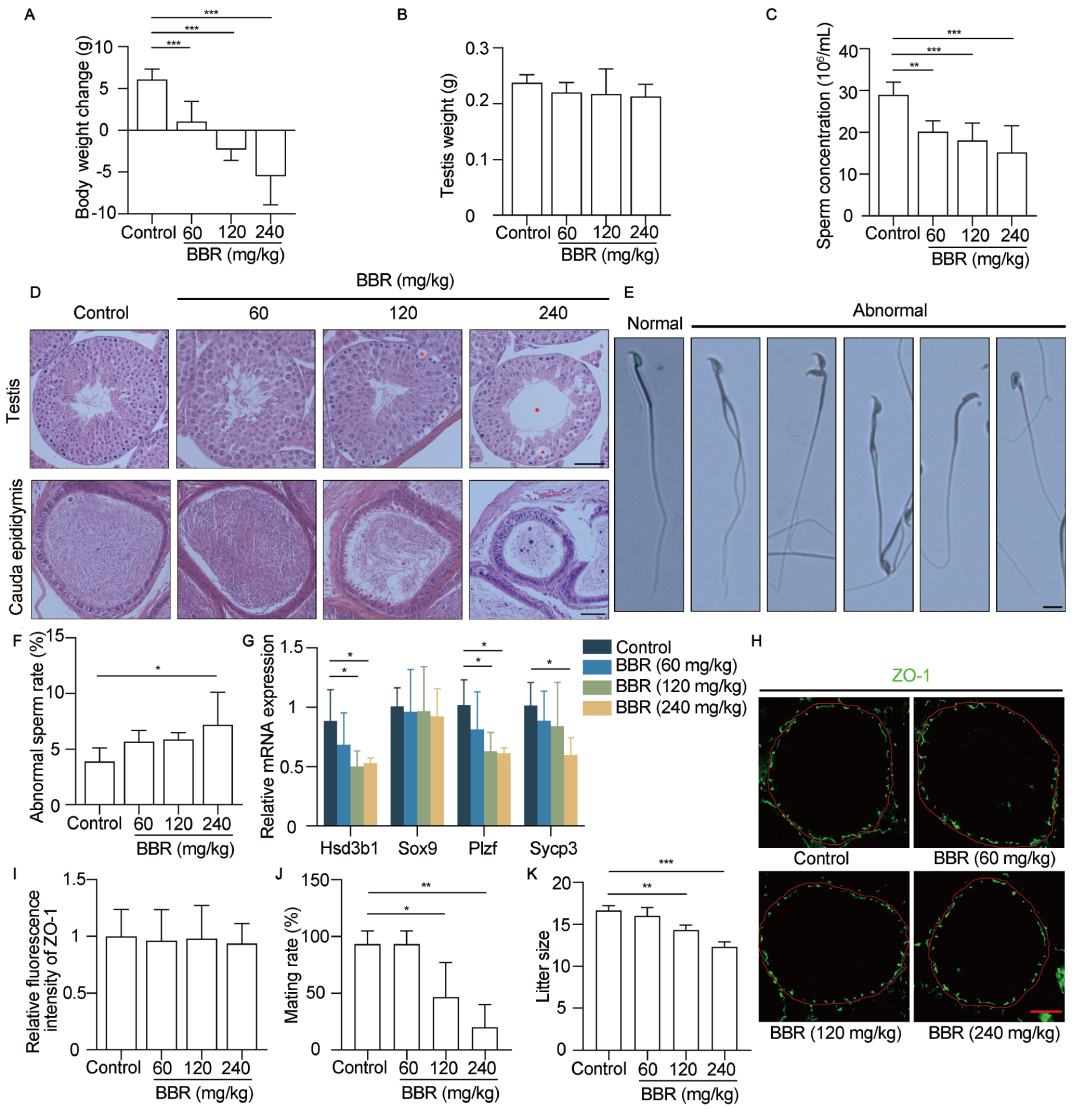
Figure 1 BBR impairs spermatogenesis and decreases the mating rate and fecundity in male mice (A,B) Body weight changes and testis weights of the mice were measured after 14 days of treatment with different BBR dosages. n = 7 for each group. Control, saline treatment. (C) Sperm from the cauda epididymis of each mouse was counted with a hemocytometer under a light microscope. n = 7 for each group. (D) Histological analysis of H&E-stained mouse testes and cauda epididymides. Asterisk, vacuolization of spermatogonia. Triangle, disordered arrangement caused by vacuolization of testicular cells. Pentagram, vacuolization of seminiferous tubules. Scale bar: 50 μm. (E) Morphological analysis of sperm from mice in the control and BBR-treated groups. The spread sperm were stained with Giemsa. Scale bar: 10 μm. (F) The rate of sperm deformation in the 4 groups was analyzed. n = 4 for each group. (G) The mRNA expression levels of testicular cell marker genes were measured. n = 4 for each group. (H) Immunofluorescence analysis of BTB integrity. The ZO-1 protein (green) was used to visualize the BTB of the testis. Scale bar: 50 μm. (I) The fluorescence intensity of ZO-1 was analyzed. n = 4 for each group, and 20 randomly selected round tubules per mouse were scored. (J) Mating rates were scored. n = 5 for each group. The experiments were repeated 3 times. (K) The litter sizes of the mice in the control and BBR-treated groups were determined. Data are expressed as the mean±SEM. Differences without statistical significance are not labelled; *P < 0.05, **P < 0.01, ***P < 0.001.

According to the clinical dose used for diarrhea treatment, three concentration gradient solutions with BBR were intragastrically administered daily to adult mice for 2 weeks. BBR administration caused significant dose-dependent weight loss in the mice ( Figure 1A). However, the testes weights were indistinguishable between control and BBR-treated mice ( Figure 1B). We found that sperm concentrations in the epididymis decreased significantly with increasing BBR dosage ( *P* < 0.01; Figure 1C). Histological examination of the testes and epididymis via H&E staining revealed that the testicular architecture and germinal cell arrangement of the mice in the control group and low-dose group were indistinguishable ( Figure 1D). However, mice in the medium-dose and high-dose groups presented disordered arrangement and vacuolization in the seminiferous epithelium of the testes, which was even worse in the high-dose groups ( Figure 1D). As the BBR dose increased, the sperm density in the mouse epididymis significantly decreased ( Figure 1D). Mice exposed to a higher dose of BBR had a greater rate of sperm deformity in the epididymis ( *P* < 0.01; Figure 1E,F). As shown in Figure 1E, the sperm deformities included double tails, double heads, double necks, banana-shaped heads, and irregular heads. In addition to those of the Sertoli cell gene *Sox9*, the mRNA levels of the Leydig cell gene *Hsd3b1*, the spermatogonial gene *Plzf* and the spermatocyte gene *Sycp3*decreased with increasing BBR dose ( Figure 1G). These results showed that the process of spermatogenesis was impaired. The blood-testis barrier (BTB), as a gatekeeper, protects the developing germ cells of males [29]. Immunofluorescence staining of ZO-1 revealed that the tight junction was not disrupted in any of the groups, and the fluorescence intensity of ZO-1 was similar in the mice treated with different BBR doses, which indicated that BBR impaired spermatogenesis but did not destroy the BTB ( Figure 1H,I). Mating behavior examination revealed that the mating rate of the mice in the medium and high groups decreased significantly due to decreased testosterone levels ( Figure 1J). To address whether BBR affects fertility, BBR-treated mice were mated with adult females, and litter sizes were recorded. The results revealed that male mice treated with BBR were fertile, but the litter sizes of the mice in the medium-dose and high-dose groups were lower than those in the control group ( Figure 1K). Taken together, these results revealed that BBR administration disrupted spermatogenesis and reduced fertility in male mice.

### TP partially reverses the damage caused by BBR to the male reproductive system

**Figure 2:**
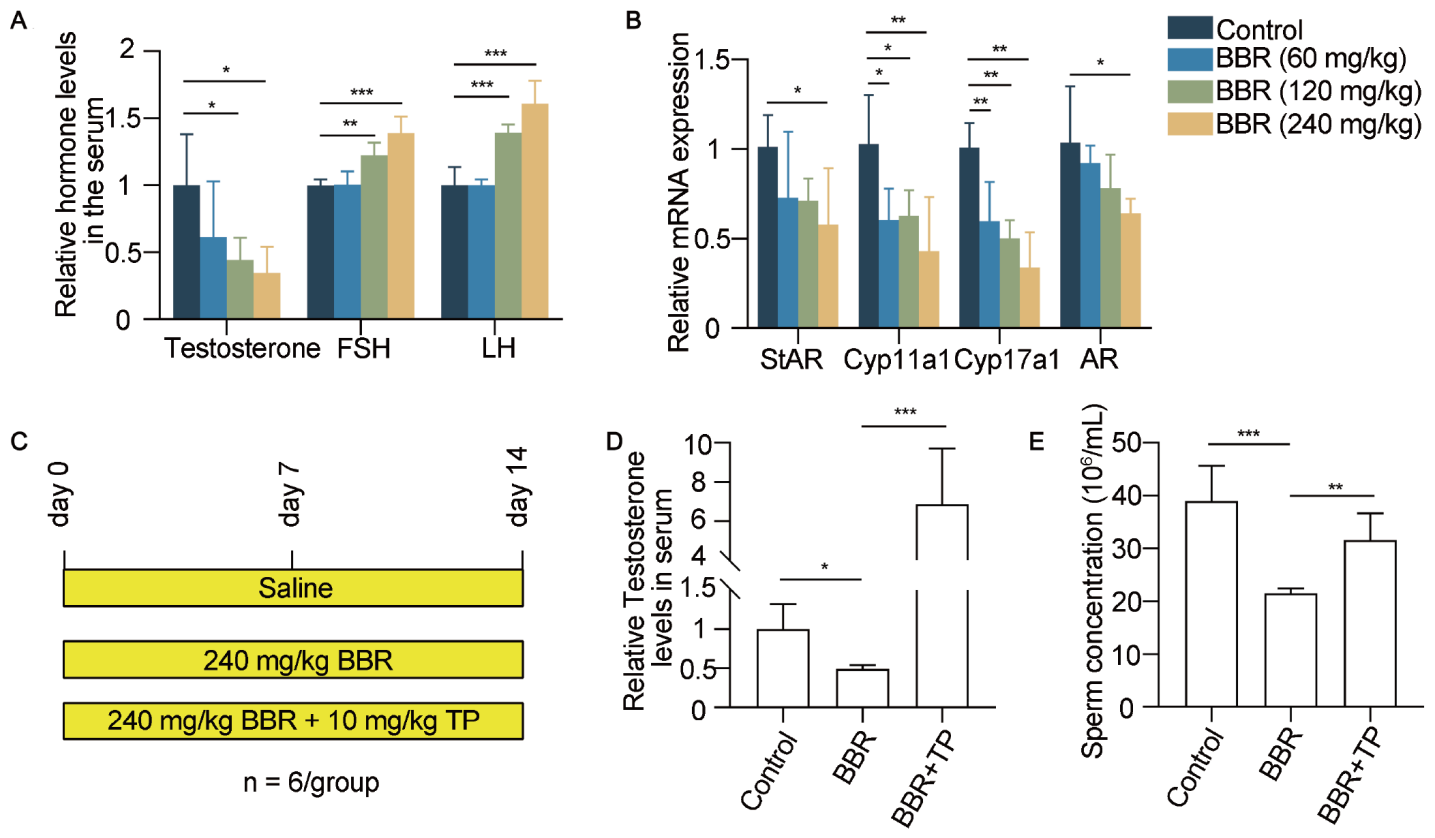
Figure 2 Testicular treatment partially reverses the damage caused by BBR to the male reproductive system (A) Changes in hormone levels related to testicular function in mice after BBR treatment. n = 4 for each group. (B) mRNA expression levels of genes related to testosterone synthesis and AR. n = 4 for each group. (C) Schematic diagram of the testicular treatment experiment. (D) The serum testosterone levels in control, BBR-treated, and BBR + TP-treated mice were measured by ELISA. n = 4 for each group. (E) The sperm concentrations were evaluated with a hemocytometer under a light microscope. n = 6 for each group. Data are expressed as the mean ± SEM. Differences without statistical significance are not labelled; *P < 0.05, **P < 0.01, ***P < 0.001.

To identify the effects of BBR on testicular function-related hormone levels, ELISA for testosterone, FSH and LH were performed. The testosterone levels in the serum decreased, and the FSH and LH levels in the serum increased with increasing BBR dosage ( Figure 2A). This phenomenon is attributed to the decrease in testosterone level caused by BBR treatment, which in turn stimulates the synthesis of gonadotropins through a feedback mechanism. Moreover, the mRNA levels of *StAR*, *Cyp11a1* and *Cyp17a1*, which are related to testosterone synthesis, were inversely correlated with increasing BBR dosage ( Figure 2B). In response to these changes, the expression of the androgen receptor (AR) also decreased ( Figure 2B). Testosterone propionate (TP), an androgen-supplementing drug, is used to treat male hypogonadism clinically. It has been reported that TP relieves the reproductive toxicity of endosulfan and vanadium to male mice [ 27, 28 ]. After 2 weeks of combined treatment with BBR and TP, the testosterone level of the mice substantially increased compared with that of the control or BBR-treated mice ( Figure 2C,D). In addition, combined administration of BBR and TP reversed the decreased sperm concentration in the mice ( Figure 2E). These results indicated that the BBR-induced decrease in sperm concentration is due to decreased testosterone synthesis in Leydig cells and that testicular treatment can partially reverse the damage caused by BBR to the male reproductive system.

### BBR administration alters the gut microbiota composition

**Figure 3:**
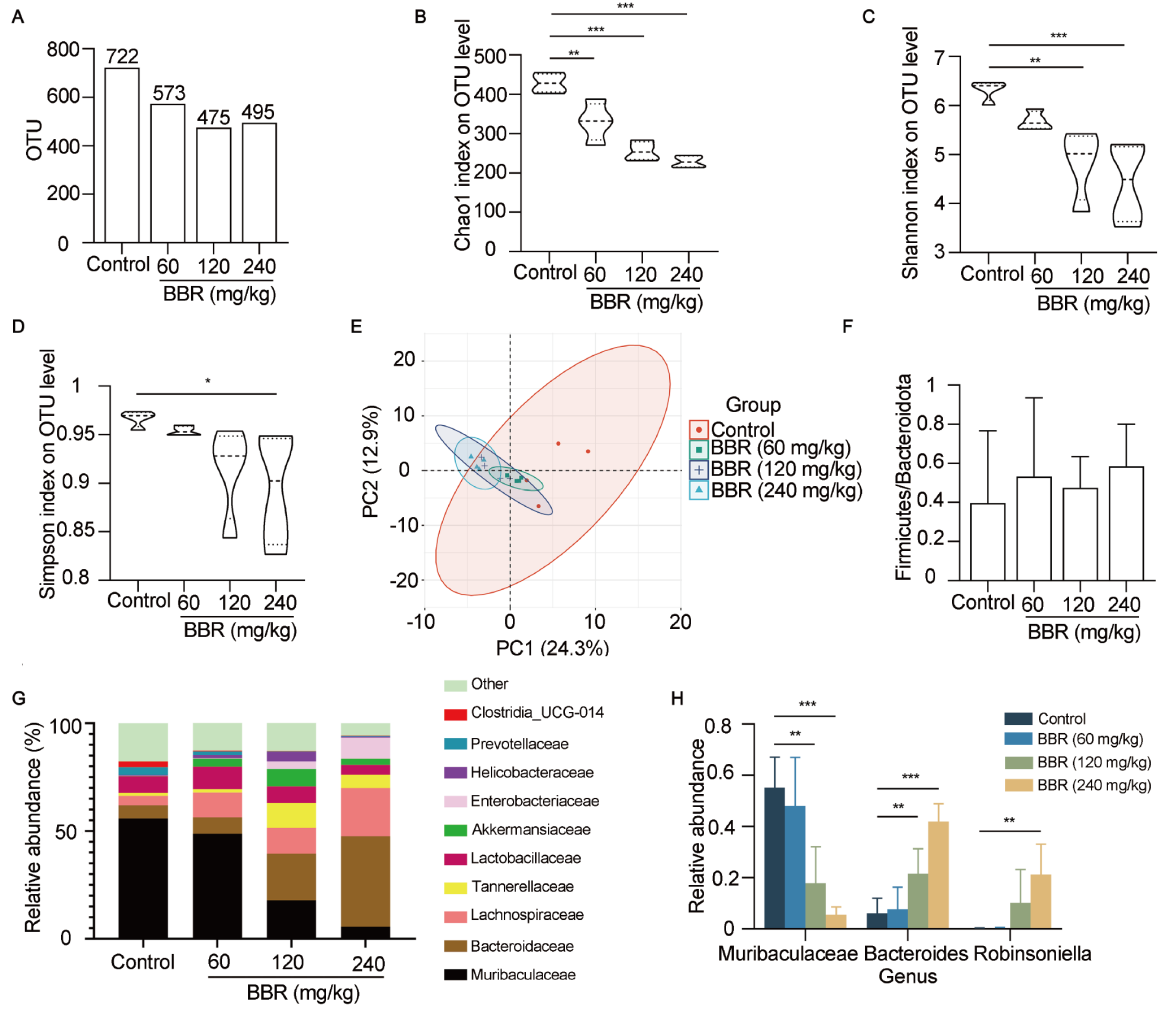
Figure 3 BBR alteres the composition of the gut microbiota in mice (A) The total fecal bacterial load and category were analyzed in mice treated with different BBR dosages. n = 6 for each group. (B–D) The Chao1 index (B), Shannon index (C) and Simpson index (D) were analyzed. n = 6 for each group. (E) PCA plot generated on the basis of the similarity of the samples in the untreated and BBR-treated groups. n = 6 for each group. (F) The Firmicutes/Bacteroidetes ratios were analyzed at the phylum level. n = 6 for each group. (G) The relative taxonomic abundances of the gut microbiota at the family level are shown in the histogram. n = 6 for each group. (H) The relative abundances of the dominant bacteria at the genus level were analyzed. n = 6 for each group. Data are expressed as the mean ± SEM. Differences without statistical significance are not labelled; *P < 0.05, **P < 0.01, ***P < 0.001.

The variation in the gut microbiota of mice was investigated because of the poor absorption and low bioavailability of BBR [30]. We sequenced the bacterial 16S rRNA gene in the feces. The sparse curves of the bacterial communities reached a saturation plateau, indicating a sufficient sequencing depth for detecting the majority of the gut bacteria ( Supplementary Figure S1). The total bacterial load or category decreased slightly with increasing BBR dosage ( Figure 3A). In addition, the Chao1 index, which represents gut microbiota richness, and the Shannon and Simpson indices, which represent diversity, decreased with increasing BBR dosage according to microbial alpha analysis ( Figure 3B‒D). Microbial beta diversity analysis based on principal component analysis (PCA) revealed obvious differences in the gut microbiota composition between the control and BBR-treated mice ( Figure 3E). The ratios of Firmicutes to Bacteroidota, an index of dysbiosis of the gut microbiota, were similar ( Figure 3F). However, BBR significantly altered the abundance of the bacterial families ( Figure 3G). The predominant bacteria in the control group were Muribaculaceae, Lactobacillaceae, and Bacteroidaceae. In contrast, the predominant bacteria in the high-dose group were Bacteroidaceae, Lachnospiraceae, and Enterobacteriaceae. Notably, the relative abundance of Muribaculaceae decreased from 55.77% to 5.63% in the high-dose group. We then analyzed the changes in the abundance of the dominant bacteria at the genus level. Depletion of Muribaculaceae and enrichment of Bacteroides were observed in the medium-dose and high-dose groups, and the high dose of BBR substantially enriched the abundance of *Robinsoniella* ( Figure 3H). These data revealed that BBR altered the composition and relative abundance of the gut microbiota, suggesting that alterations in the composition of the gut microbiota disrupted spermatogenesis in mice.

### Muribaculaceae is responsible for the effects of BBR on spermatogenesis in mice

**Figure 4:**
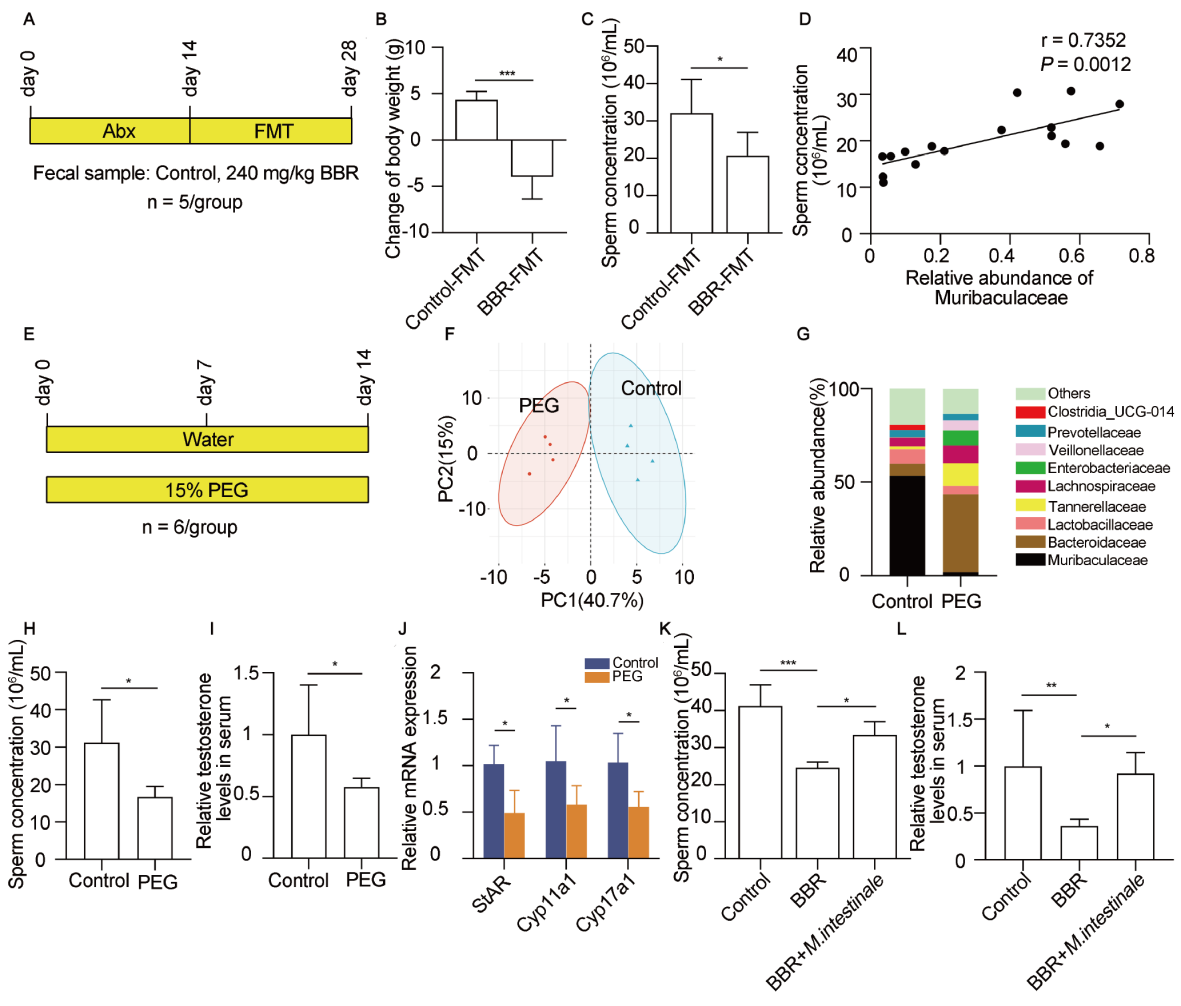
Figure 4 Muribaculaceae abundance regulates spermatogenesis in mice (A) Schematic diagram of the FMT experiment. Abx, a cocktail of broad-spectrum antibiotics. n = 5 for each group. (B) Statistical analysis of changes in body weight after FMT. Control-FMT, the group of mice transplanted with feces from control mice; BBR-FMT, the group of mice transplanted with feces from BBR-treated mice. n = 5 for each group. (C) Sperm concentrations from the cauda epididymides of the mice were analyzed with a hemocytometer under a light microscope. n = 5 for each group. (D) The correlation between Muribaculaceae abundance and sperm concentration was analyzed in the control, low-dose, medium-dose and high-dose groups. n = 4 for each group. (E) Schematic diagram of the PEG treatment experiment. n = 6 for each group. (F) PCA plot generated on the basis of the similarity of fecal samples in the control and PEG-treated groups. (G) The relative taxonomic abundances of the gut microbiota at the family level are shown in the histogram. (H) Sperm from the cauda epididymides of the mice were counted with a hemocytometer under a light microscope. n = 6 for each group. (I) The serum testosterone concentration in untreated and PEG-treated mice was measured via ELISA. n = 5 for each group. (J) The mRNA expression levels of genes related to testosterone synthesis were measured by RT-qPCR. n = 4 for each group. (K) The sperm concentrations of untreated, BBR-treated, and BBR+M. intestinale-treated mice were evaluated with a hemocytometer under a light microscope. n = 5 for each group. (L) The testosterone levels in mouse serum were measured by ELISA. n = 5 for each group. Data are expressed as the mean ± SEM. Differences without statistical significance are not labelled; *P < 0.05, **P < 0.01, ***P < 0.001.

To investigate the effect of the gut microbiota on spermatogenesis, mice pretreated for gut microbiota reconstitution with an antibiotic cocktail were subjected to FMT with BBR-treated or control donors ( Figure 4A). Body weight and epididymal sperm concentration were significantly reduced in BBR-FMT mice, strongly indicating that the gut microbiota regulated spermatogenesis in these mice ( Figure 4B,C). 16S rRNA sequencing of the gut microbiota revealed a drastic decrease in Muribaculaceae abundance in BBR-FMT mice, followed by a correlation between Muribaculaceae abundance and sperm concentration in mice ( Figure 4D). As expected, the relative abundance of Muribaculaceae was strongly correlated with the sperm concentration (r = 0.7352, *P* = 0.0012). As polyethylene glycol (PEG) can alter intestinal osmolality and deplete Muribaculaceae abundance [17], we administered PEG to examine the role of Muribaculaceae in spermatogenesis ( Figure 4E). The total bacterial abundance or taxa in the feces decreased significantly after PEG treatment ( Supplementary Figure S2A‒D). PCA of microbial beta diversity revealed obvious differences in the gut microbiota composition between the control and PEG treatment groups ( Figure 4F). Although the richness and diversity of microbes were reduced in the intestines of PEG-treated mice, the ratios of Firmicutes to Bacteroidota were similar between control and PEG-treated mice ( Supplementary Figure S2E). As expected, the dominant microbiota of the intestine in the mice treated with PEG was converted from Muribaculaceae to Bacteroidaceae at the family level, and the relative abundance of Muribaculaceae decreased from 53.42% to 1.79% ( Figure 4G and Supplementary Figure S2F). Histological examination revealed that the mice in the PEG-treated group presented a lower epididymal sperm density, decreasing by 46.73% ( Figure 4H). To determine the upstream factor causing sperm reduction, we collected serum from PEG-treated and control mice to measure testosterone levels. Like that in BBR-treated mice, the serum testosterone level in the PEG-treated group decreased by 42.27% compared with that in the control group ( Figure 4I). In addition, the mRNA levels of *StAR*, *Cyp11a1* and *Cyp17a1* in the testes were significantly decreased in the PEG-treated mice ( Figure 4J). Thus, our data demonstrated that a low abundance of Muribaculaceae compromised sperm production by regulating testosterone level.

The irreversible decrease in Muribaculaceae abundance prompted us to ask whether spermatogenesis disruption resulted from Muribaculaceae depletion is reversible. Among the ten species in the Muribaculaceae family, the *M*. *intestinale*, which is representative of four isolates, was cultured anaerobically *in vitro* ( Supplementary Figure S3) [31]. *M* . *intestinale* culture medium was intragastrically administered to the mice for 14 days together with BBR, and the sperm concentration and testosterone level were measured. As expected, the administration of *M* . *intestinale* reversed the decrease in the sperm concentration in the mice treated with BBR by 88.11% ( Figure 4K). Moreover, testosterone level also recovered to 92.23% in the mice treated with BBR and *M*. *intestinale* compared with that in the untreated mice ( Figure 4L). These data suggest that *M*. *intestinale* administration can prevent the disruption caused by BBR during spermatogenesis.

### BBR alters gut microbiota metabolism

**Figure 5:**
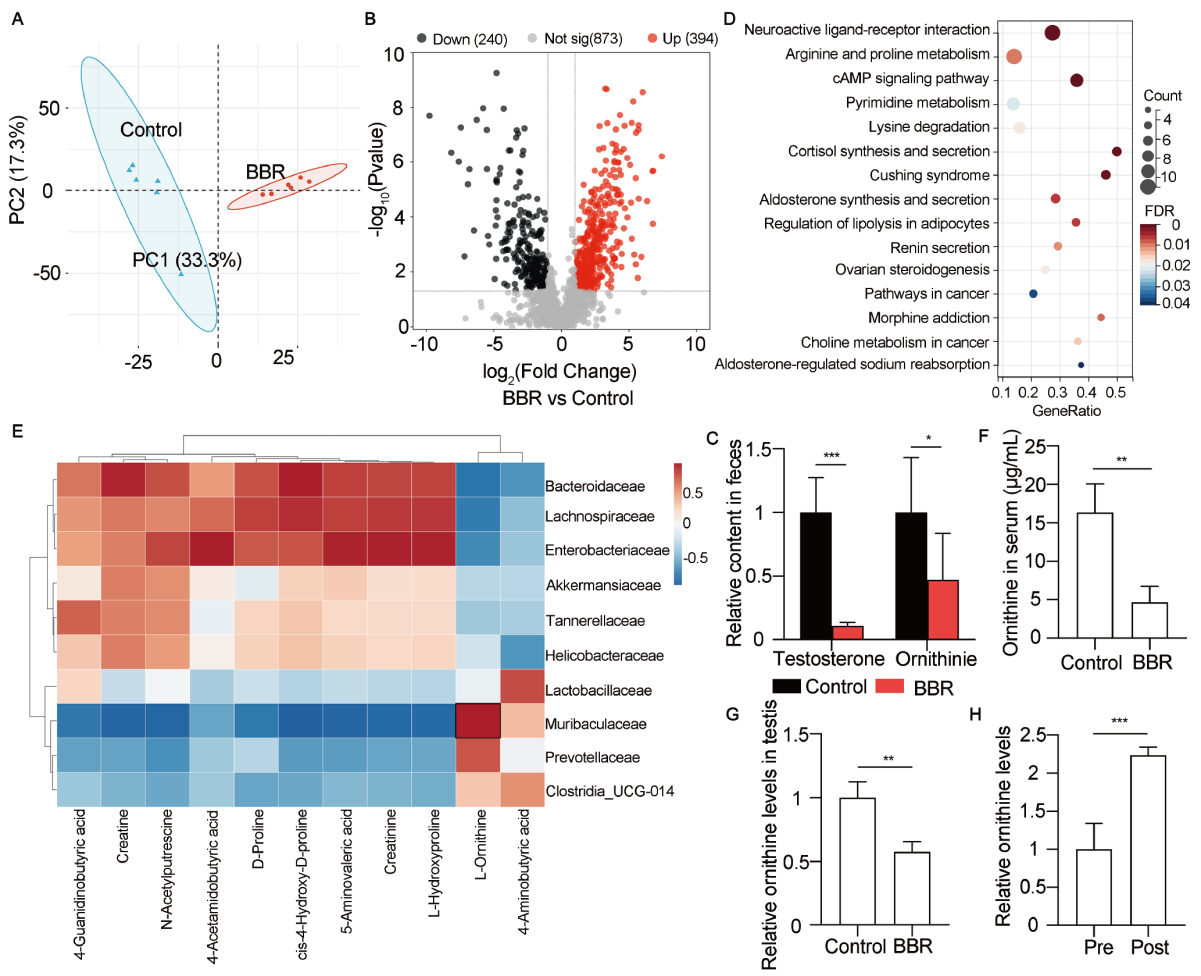
Figure 5 BBR affects ornithine metabolism in the mouse gut microbiota (A) PCA plot was generated on the basis of the similarity of the samples in the control and BBR-treated (high-dose) groups. n = 6 for each group. (B) The differentially abundant metabolites of the gut microbiota are shown in a volcano plot. P < 0.05. (C) The testosterone (n = 6 for each group) and ornithine contents (n = 4 for each group) in the fecal samples were analyzed according to the LC-MS/MS-based metabolomics results. (D) Significant KEGG pathways associated with the fecal microbiome of the untreated and BBR-treated groups were identified. (E) The close correlations between bacterial abundance at the family level and the differentially abundant metabolites of the arginine and proline metabolism pathways were analyzed in the control and BBR-treated groups. n = 4 for each group. (F) The ornithine level in the serum was analyzed by high-performance liquid chromatography (HPLC). n = 4 for each group. (G) The ornithine levels in the testes were measured by LC-MS/MS. n = 4 for each group. (H) The ornithine levels in the media with or without M. intestinale were measured by LC-MS/MS. M. intestinale was cultured for 72 h in vitro. Pre, precultured. Post, postculture. n = 6 for each group. Data are expressed as the mean ± SEM. Differences without statistical significance are not labelled; *P < 0.05, **P < 0.01, ***P < 0.001.

Since gut microbiota metabolites regulate the biological processes of the host, we sought to determine whether metabolite changes caused by Muribaculaceae disorders affect testosterone level. We analyzed the metabolic features of the gut microbiota of the mice in the control and BBR-treated groups by LC-MS/MS. PCA score plots revealed that the high-dose BBR-treated group was clearly separated from the control group ( Figure 5A). We then compared the fecal metabolomics profiles of both groups and identified 394 upregulated metabolites and 240 downregulated metabolites in the BBR-treated group compared with the control group ( Figure 5B). Like the serum testosterone level, the level of fecal testosterone was also decreased by 89.17% compared with that in the controls, suggesting that testosterone synthesis from Leydig cells was disrupted ( Figure 5C). Differentially abundant metabolites were enriched to identify the biological pathways involved, including neuroactive ligand-receptor interaction, arginine and proline metabolism, the cAMP signaling pathway, pyrimidine metabolism, and lysine degradation ( Figure 5D). Interestingly, Muribaculaceae is related to the ornithine biosynthesis pathway according to the annotation of bacterial genomes [32]. As ornithine is an indispensable metabolite in the arginine and proline metabolism pathways, we explored the correlation between Muribaculaceae and ornithine. We performed correlation analysis between the top 10 microbes at the family level and the differentially abundant metabolites of the arginine and proline metabolism pathways ( Figure 5E). The results showed that Muribaculaceae was highly correlated with metabolites involved in the arginine and proline metabolism pathways, including 4-guanidinobutyric acid (r = ‒0.7406), creatine (r = ‒0.9344), N-acetylputrescine (r = ‒0.8534), 4-acetamidobutyric acid (r = ‒0.5857), D-proline (r = ‒0.7701), cis-4-hydroxy-D-proline (r = ‒0.9467), 5-aminovaleric acid (r = ‒0.9013), creatinine (r = ‒0.8688), L-hydroxyproline (r = ‒0.8694), L-ornithine (r = 0.9313) and 4-aminobutyric acid (r = 0.5035). In addition, Muribaculaceae and L-ornithine were strongly positively correlated, and ornithine levels in the feces, serum and testes of the mice in the BBR-treated group decreased drastically ( Figure 5C,F,G). Metabolite analysis of the culture medium before and after cultivation of *M* . *intestinale* revealed a 2.23-fold increase in the level of ornithine after cultivation ( Figure 5H). Overall, these data showed that Muribaculaceae regulated ornithine synthesis.

### BBR influences the steroid metabolic process

**Figure 6:**
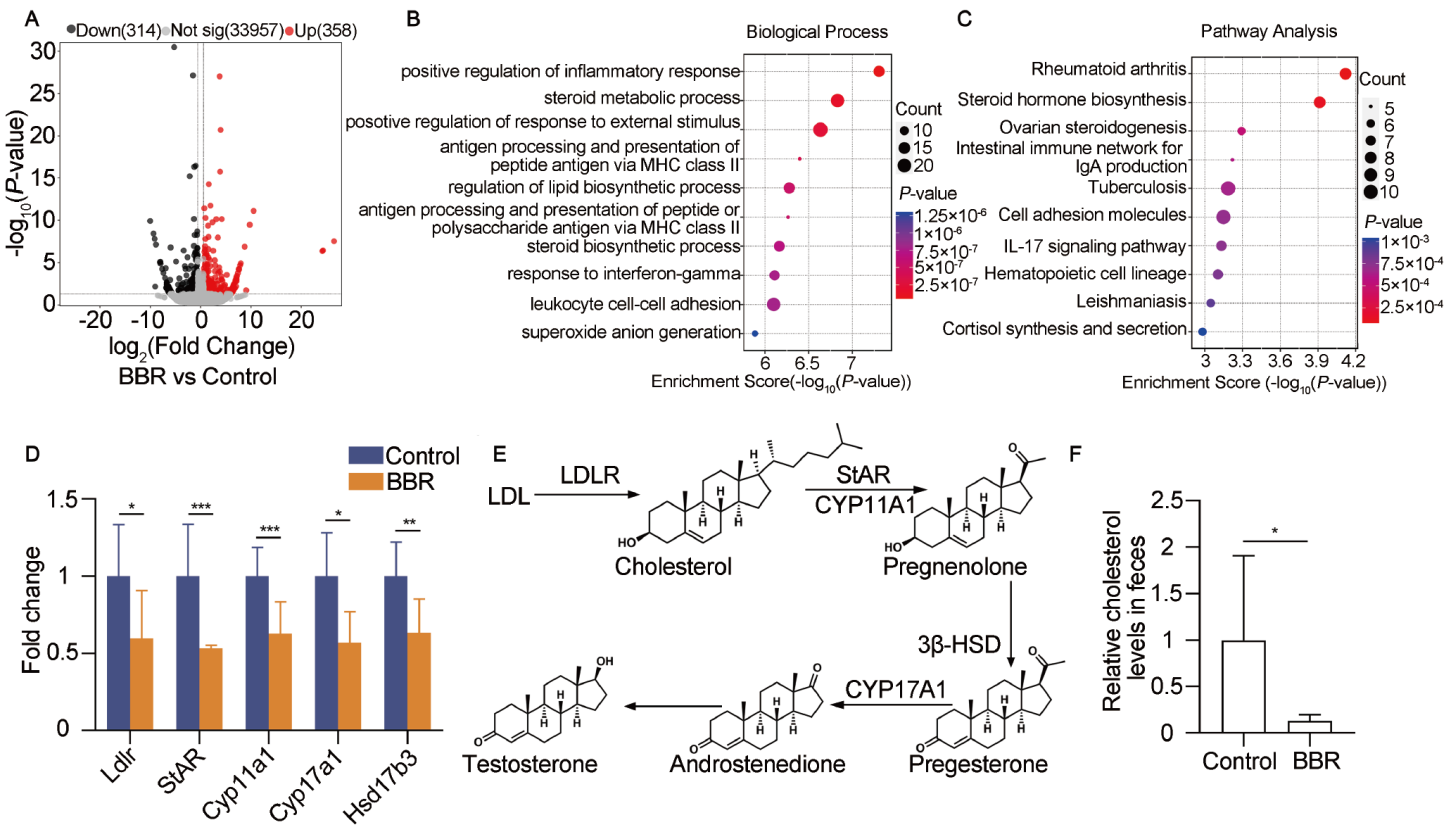
Figure 6 BBR affects the steroid metabolism related to *Ldlr* in mouse testes (A) DEGs between control and BBR-treated (high-dose) mice were analyzed. P < 0.05. (B) The biological processes associated with the DEGs were analyzed through GO analysis. (C) KEGG pathways associated with the DEGs were identified. (D) The mRNA levels of Ldlr-associated genes related to testosterone synthesis were analyzed. n = 2 for each group. (E) The LDLR-induced testosterone synthesis pathway is shown. (F) The cholesterol levels in the feces were analyzed. n = 6 for each group. Data are expressed as the mean ± SEM. Differences without statistical significance are not labelled; *P < 0.05, **P < 0.01, *** P < 0.001.

To further delineate the mechanism by which the metabolite ornithine regulates spermatogenesis, we collected whole-testis samples for RNA sequencing. A total of 672 differentially expressed genes (DEGs) were identified, with more than 1.5-fold differences between the high-dose BBR-treated group and the control group. Among them, 314 genes were downregulated, and 358 genes were upregulated after BBR treatment ( Figure 6A). GO analysis of these DEGs revealed a series of biological processes, such as positive regulation of the inflammatory response, positive regulation of the response to external stimuli, antigen processing and presentation of peptide antigens via MHC class II, and regulation of lipid biosynthetic processes, particularly testosterone synthesis-related steroid metabolic processes ( Figure 6B). Molecular function analysis also revealed that these DEGs are associated with the function of steroid binding, which is related to testosterone synthesis ( Supplementary Figure S4A,B). KEGG pathway analysis revealed a series of pathways, such as rheumatoid arthritis, steroid hormone biosynthesis, ovarian steroidogenesis, the intestinal immune network for IgA production and tuberculosis ( Figure 6C). Among these, steroid hormone biosynthesis is critical for testosterone synthesis. In particular, a series of testosterone synthesis-related genes, including *Ldlr*, *StAR*, *Cyp11a1*, *Cyp17a1* and *Hsd17b3*, were significantly downregulated ( Figure 6D). Testosterone synthesis in Leydig cells is regulated mainly by the *Ldlr gene* ( Figure 6E). LDLR transports low-density lipoprotein into Leydig cells to participate in cholesterol synthesis and regulate cholesterol homeostasis, which is a critical substrate for testosterone synthesis [ 33 , 34]. Importantly, the cholesterol level in the feces of the BBR-treated mice was drastically lower than that in the feces of the control mice ( Figure 6F). On the basis of the above findings, we speculated that *Ldlr* is a potential target of the metabolite ornithine for maintaining cholesterol and testosterone synthesis.

### Ornithine participates in steroid metabolism by regulating *Ldlr* transcription

**Figure 7:**
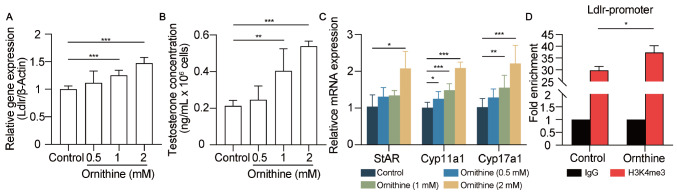
Figure 7 Promotion of *Ldlr* promoter accessibility by ornithine (A) Ldlr mRNA level was measured by RT-qPCR. n = 5 for each group. (B) The testosterone levels in the medium were measured after the TM3 cells were treated with different concentrations of ornithine (0. 0.5, 1 and 2 mM). n = 5 for each group. (C) The mRNA expression levels of genes related to testosterone synthesis were measured by RT-qPCR. n = 5 for each group. (D) ChIP-qPCR analysis of the direct binding of H3K4me3 to the Ldlr promoter was performed. IgG was used as the negative control. n = 3 for each group. Data are presented as the mean ± SEM. Differences without statistical significance are not labelled; *P < 0.05, **P < 0.01, ***P < 0.001.

Next, we explored how ornithine regulates *Ldlr* expression in TM3 cells, a mouse Leydig cell line. We found that the expression of the *Ldlr* gene significantly increased with increasing ornithine dosage ( Figure 7A). Moreover, the levels of testosterone and testosterone synthesis-related gene expression increased as the ornithine concentration increased ( Figure 7B,C). These data indicate that ornithine facilitates the expression of *Ldlr* to increase testosterone level. Histone modifications regulate gene expression by altering chromatin accessibility. Histone H3 lysine 4 trimethylation (H3K4me3) and histone H3 lysine 27 trimethylation (H3K27me3) are associated with active and repressive transcription, respectively [35]. Therefore, we performed ChIP-qPCR and analyzed the H3K4me3 occupancy of the *Ldlr*promoter. Quantitative analysis revealed a 29.77-fold enrichment of H3K4me3 at the *Ldlr* promoter compared with that of IgG ( Figure 7D). We also found a 1.25-fold increase in H3K4me3 occupancy in the target region in the ornithine-treated group compared with the control group ( Figure 7D), suggesting that ornithine increased the promoter accessibility of *Ldlr* and promoted LDLR expression.

## Discussion

In this study, we revealed that the dysbiosis of the gut microbiota induced by BBR is one of the primary causes of impaired spermatogenesis in mouse hosts. BBR alters the gut microbiota composition, leading to a drastic decrease in the abundance of Muribaculaceae. Combined analysis of gut microbiota sequencing and metabolomics revealed that Muribaculaceae regulates ornithine level. A decrease in ornithine level leads to lower chromatin accessibility on the *Ldlr* promoter. Therefore, low-density lipoprotein cannot be transported by LDLR into the cytoplasm of Leydig cells for the intracellular synthesis of cholesterol, resulting in impaired testosterone synthesis and hence impaired spermatogenesis. These results suggest the potential harm of BBR on adult couples trying to conceive, as well as on the fertility of male patients who are prescribed BBR-containing medications.

Notably, BBR reportedly improves sperm motility and quantity in diabetic rats [36]. However, these reports do not directly contradict our conclusions. Diabetes is a metabolic disease characterized by hyperglycemia, which promotes the overproduction of oxidative molecules, resulting in histopathological alterations of the seminiferous epithelium and decreases in sperm concentration and quality [37]. BBR attenuates ROS production by inhibiting the activation of the AK2/NF-κB pathway and protects reproductive function and spermatogenesis [36]. We noticed that sperm motility decreased to less than 20% in diabetic mice and recovered to 40% after BBR treatment. However, even after BBR treatment, sperm motility was still not restored to normal levels in diabetic mice. Therefore, we believe that the role of BBR is diverse. BBR prevents sperm damage by inhibiting the ROS/AK2/NF-κB pathway and disrupts spermatogenesis by reducing Muribaculaceae abundance. In addition, the abundance of Muribaculaceae in the gut of mice is approximately 50%, but it is only 20%–30% in SD rats [ 38, 39]. Since BBR works through the gut microbiota due to its poor bioavailability, these factors explain the differences in the effects of BBR on sperm quality between mice and SD rats.

Our RNA sequencing results revealed that inflammatory and immune pathways were affected, suggesting that inflammation occurred in the testes of BBR-treated mice ( Figure 6B,C). TREM2, a transmembrane receptor of the immunoglobulin superfamily, promotes cell survival and counteracts inflammation [40]. TREM2 inhibits neuroinflammation and neuronal apoptosis via the PI3K/AKT pathway after intracerebral hemorrhage in mice [41]. The RNA-seq results revealed that the *Trem2* mRNA level was significantly elevated in the testes of BBR-treated mice ( Supplementary Figure S4C). The immunofluorescence results revealed that TREM2 was expressed only in Leydig cells. The expression of TREM2 was low in untreated mice but increased dramatically after BBR treatment, which is consistent with the RNA-seq results ( Supplementary Figure S4D). Therefore, we conclude that BBR causes inflammation in the testis, which leads to a decrease in TREM2 expression to alleviate the inflammatory response.

Although BBR is a drug for treating diarrhea, diabetes, hyperlipidemia, and cancer, how it achieves its effects *in vivo* remains unknown because of its poor bioavailability after oral administration. The intestinal absorption of BBR is less than 1%, and the absorbed BBR can be excreted back to the intestinal lumen through the action of P-glycoprotein [42]. Thus, the gut microbiota is the primary pathway through which BBR functions. In this study, we found that the abundance of Muribaculaceae and the decrease in sperm concentration in mice were closely related. Considering the drastic decrease in the abundance of Muribaculaceae after BBR treatment in mice, the following explanation seems reasonable: First, BBR attenuates bactericidal activity and inhibits the growth of protozoa [43]. BBR has been shown to be bactericidal against *V*. *cholera* and protozoacidal against *Giardia lamblia* [ 44, 45]. Our results revealed a 50.14% decrease in the relative abundance of Muribaculaceae after BBR treatment, indicating that BBR may also have bactericidal activity against the Muribaculaceae family. Second, the Muribaculaceae family is highly sensitive to osmolality. Higher osmolality leads to less viable Muribaculaceae [17]. PEG, which is not absorbed by the intestinal epithelium, decreases Muribaculaceae by increasing osmolality [17]. Therefore, BBR administration may cause the disappearance of Muribaculaceae by changing the intestinal osmolality. Notably, the decrease in Muribaculaceae abundance is irreversible.

There has been little research on Muribaculaceae. In this study, we identified the biological significance of Muribaculaceae for the first time. Muribaculaceae is abundant, reaching approximately 50% at the family level in the gut of mice [17]. It was speculated to be involved in ornithine biosynthesis via genome annotation, but this hypothesis has not been validated [32]. We found a strong correlation between the sperm concentration and Muribaculaceae abundance. Combined analysis of metabolomics and 16S rRNA sequencing indicated that Muribaculaceae regulates ornithine synthesis. These results are important because diseases with decreased ornithine level can be treated via supplementation with Muribaculaceae via fecal transplantation.

Notably, the metabolite ornithine is recognized as a bridge connecting the gut microbiota and spermatogenesis via blood circulation, and the decrease in the metabolite ornithine in the gut microbiota disrupted spermatogenesis in the mice in our study. First, causal relationships between the gut microbiome and blood metabolites have been demonstrated. The metabolites of the gut microbiota affect its host. Nearly half of blood metabolites are associated with gut microbial species and their metabolites [46]. The gut microbiota mediates the transport of its metabolites through the gut barrier into the bloodstream [46]. It has been reported that the microbes harbored by the host, which convert cholesterol to the sterol coprostanol, have lower fecal cholesterol level and lower total serum cholesterol [47]. Interestingly, we also found that the ornithine levels in the feces, serum and testes of the BBR-treated mice were decreased ( Figure 5C,F,G). In addition, polyamines have been reported to be involved in cell differentiation and proliferation, and ornithine decarboxylase (ODC1) metabolizes ornithine to polyamines [48]. We found that the mRNA level of *Odc1* decreased significantly in the testes ( Supplementary Figure S5A). These results suggest that changes in the metabolites of the gut microbiota could affect tissues and organs via blood circulation.

The biosynthesis of testosterone from cholesterol in Leydig cells plays an essential role in the maintenance and regulation of male fertility. Cholesterol is metabolized to pregenenolone via the inner mitochondrial membrane of CYP11A1, which is subsequently converted to testosterone by mitochondria and smooth endoplasmic reticulum enzymes [49]. Cholesterol is derived from four sources in Leydig cells: *de novo* synthesis in the endoplasmic reticulum, utilization of cholesteryl esters in lipid droplets by cholesteryl ester hydrolase, uptake of plasma lipoprotein-derived cholesteryl esters by LDLR or SR-BI, and plasma membrane-associated free cholesterol [50]. LDLR-transported low-density lipoprotein is the main source of intracellular cholesterol synthesis [49]. We found that a decrease in LDLR expression substantially reduced cholesterol synthesis. Cold shock domain-containing protein E1 (CSDE1) facilitates the degradation of *Ldlr* mRNA [51]. However, we found no difference in the mRNA level of CSDE1 after BBR treatment, indicating that the decrease in LDLR expression is not mediated by CSDE1 ( Supplementary Figure S5B). Therefore, we speculate that ornithine modulates the transcription of *Ldlr* through unknown mechanisms on the basis of the results of H3K4me3 ChIP-qPCR, and further research is needed.

Nevertheless, this study has several limitations. We observed that BBR decreased the abundance of Muribaculaceae in the guts of the mice and that supplementation with *M*. *intestinale* partially reversed the decrease in sperm concentration caused by BBR, which implied that BBR impairs spermatogenesis not exclusively by regulating the gut microbiota composition. The direct harmful effects of BBR on spermatogenesis in male mice are still unknown. In our future work, we intend to focus on other regulatory pathways of BBR in spermatogenesis in addition to the gut microbiota. In addition, it is necessary to explore the regulatory mechanism of ornithine on *Ldlr* transcription.

## Availability of Data and Materials

All data relevant to the study are included in the article or are uploaded as supplementary information. All the raw sequences have been deposited in the NCBI Sequence Read Archive (SRA) database ( https://www.ncbi.nlm.nih.gov/) with the following accession numbers: PRJNA978974 (RNA sequencing of testes), PRJNA898107 (16S-rRNA sequencing of BBR feces), and PRJNA989049 (16S-rRNA sequencing of PEG feces).

## Supporting information

24196Supplementry_Data

## Supplementary Data

Supplementary Data is available at *Acta Biochimica et Biophysica Sinica* online.
