# Supplementary material for: Berberine alters the gut microbiota metabolism and impairs spermatogenesis: Berberine alters gut microbiota metabolism and impairs spermatogenesis
Source: Acta Biochim Biophys Sin (Shanghai). 2024 Oct 15;57(4):569–81. doi: 10.3724/abbs.2024174 (PMC12040761; doi:10.3724/abbs.2024174)
Supplement: 24196Supplementry_Data [file 24196Supplementry_Data.docx]

**Supplementary Table S1. The information of antibodies used for immunofluorescene staining**

| Name | Cat. # | Source | Company |
| --- | --- | --- | --- |
| ZO-1 | 21773-1-AP | Rabbit | Proteintech |
| TREM2 | A10482 | Rabbit | ABclonal |
| CoraLite488 goat anti-rabbit IgG | SA00013-2 | Goat | Proteintech |
| DAPI | ZLI-9557 | - | ZSGB-BIO |
| Control IgG | AC005 | Rabbit | ABclonal |
| H3K4me3 | Ab8580 | Rabbit | ABCAM |

**Supplementary Table S2. Primer sequences used for qRT-PCR of the target genes**

| Gene | Primer sequence (5′→3′) | Amplicon (bp) |
| --- | --- | --- |
| *Plzf* | F: CTGGACAGTTTGCGACTGAG | 123 bp |
|  | R: GTCTGTGTGTCTCCA |  |
| *Hsd3b1* | F: TGTCACAGGTGTCATTCCCA | 113 bp |
|  | R: AGCTGCAGAAGATGAAGGC |  |
| *Sycp3* | F: GGACAGCGACAGCTCAC | 100 bp |
|  | R: ATCAACCAAAGGTGGCTTCC |  |
| *Sox9* | F: AGCTGGCAAAGTTGATCTG | 136 bp |
|  | R: GACGTCGAAGGTCTCAATGTTGG |  |
| *Cyp11a1* | F: AGGAGACACTGAGACTCCAC | 132 bp |
|  | R: GATCTCGACCCATGGCAAAG |  |
| *Cyp17a1* | F: AATGAATGGGACCAGCCAGA | 93 bp |
|  | R: GGGCAAATAACTGGGTGTGG |  |
| *StAR* | F: CAGGAAGGCTGGAAGAAGGA | 199 bp |
|  | R: TCTGCAGGACCTTGATCTCC |  |
| *β-actin* | F: GAGACCTTCAACACCCCAGCC | 362 bp |
|  | R: CCGTCAGGCAGCTCATAGCTC |  |
| *Ldlr* | F：TCCACTGTGGTAGCAGTGAG | 146 bp |
|  | F：GTGAATGCAGGAGCCATCTG |  |
| *Ldlr-pro* | F: TGGAAACCTCGCCCCTAGTA | 140 bp |
|  | R: AATGTTCCCGCTGCAAACAC |  |
| *AR* | F: GGCGGTCCTTCACTAATGTCAACT | 79 bp |
|  | R: TGTGCATGCGGTACTCATTG |  |

**
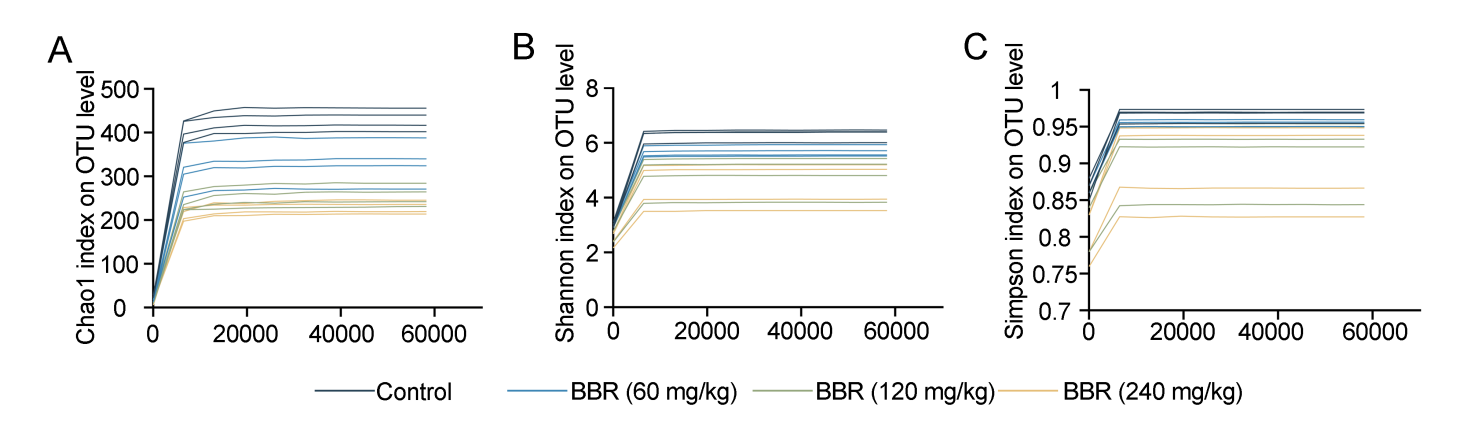
**

**Supplementary Figure S1. The rarefaction curves of the bacterial community**  (A‒C) The rarefaction curves of Chao1 index (A), Shannon index (B) and Simpson index (C) are shown.

**
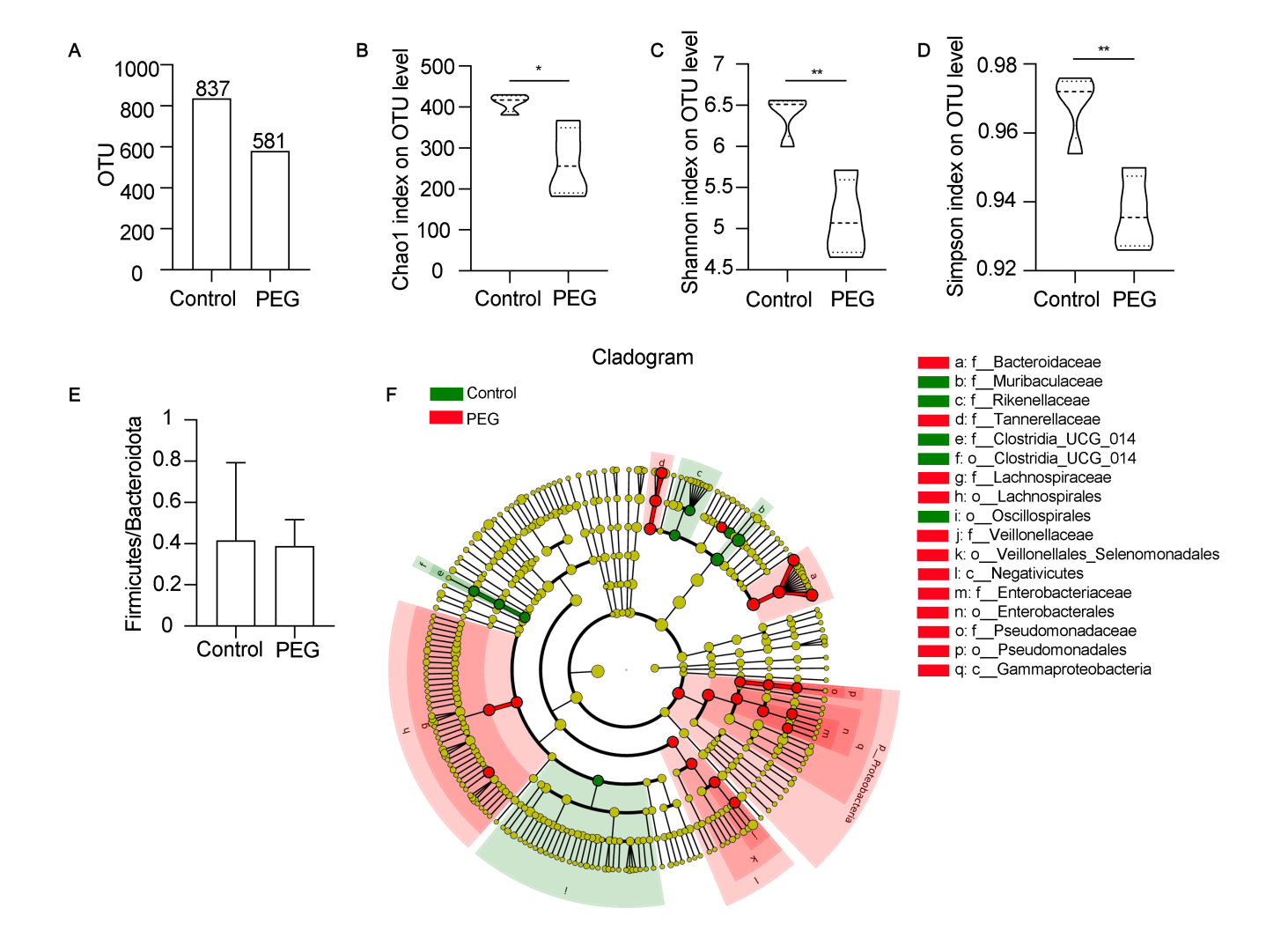
**

**Supplementary Figure S2. PEG inhibits the *Muribaculaceae*** (A) The total fecal bacterial load and category were analyzed in control and PEG-treated groups. (B‒D) The alpha diversity indices (Chao1 index (B), Shannon index (C) and Simpson index (D)) were analyzed. *n*=4 for each group. (E) The ratios of Firmicutes/Bacteroidota were analyzed at the phylum level. *n*=4 for each group. (F) Taxonomic cladogram from linear discriminant analysis effect size (LEfSe) was shown. Dot size is proportional to the abundance of the taxon. *n*=4 for each group. Data are expressed as the mean ± SEM. Differences without statistical significance were not labeled; **P* < 0.05, ***P* < 0.01, ****P* < 0.001.

**
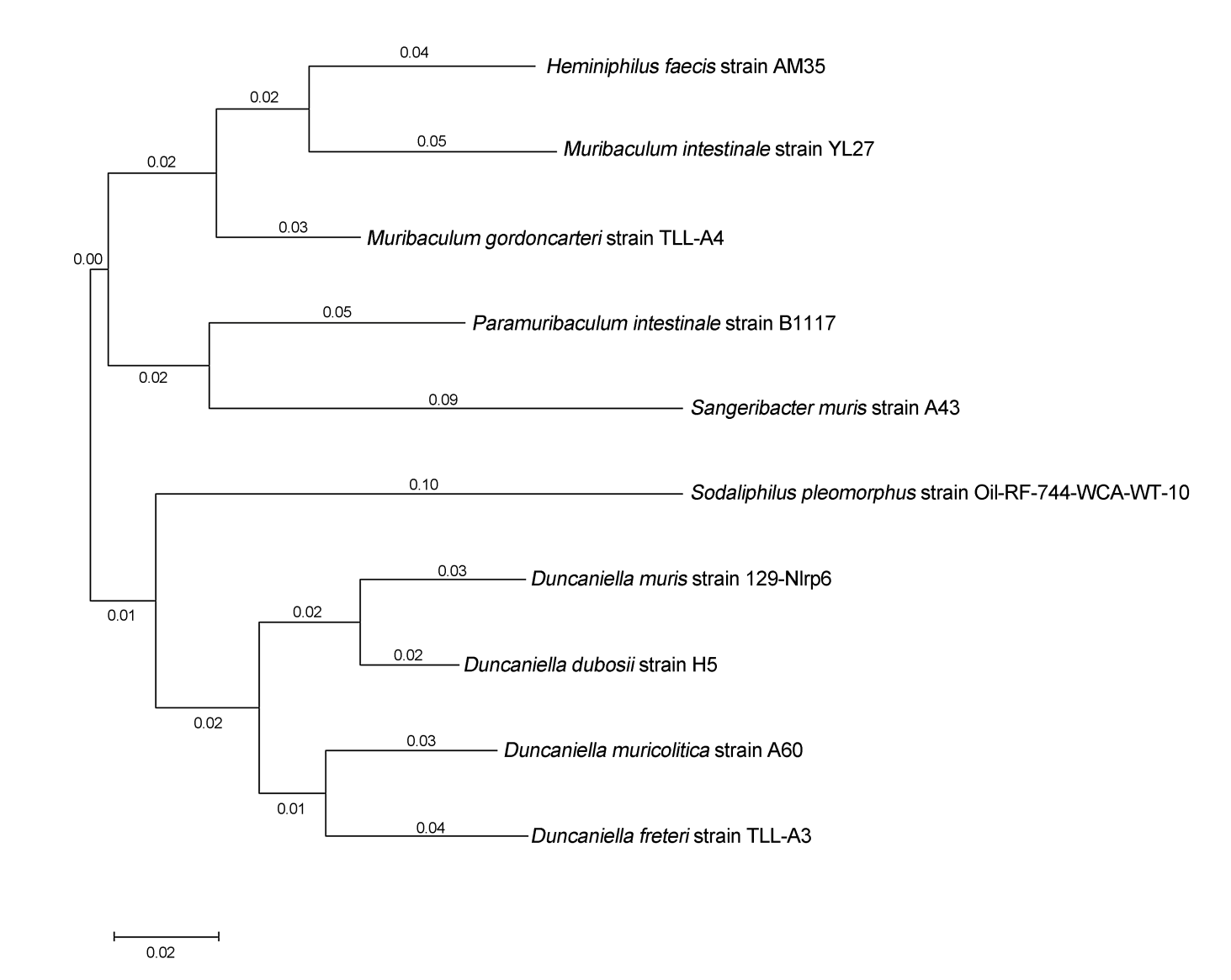
**

**Supplementary Figure S3. Phylogenetic tree showing the relation of *Muribaculaceae* isolates** Bar, 0.02 indicates nucleotide substitutions per position.

**
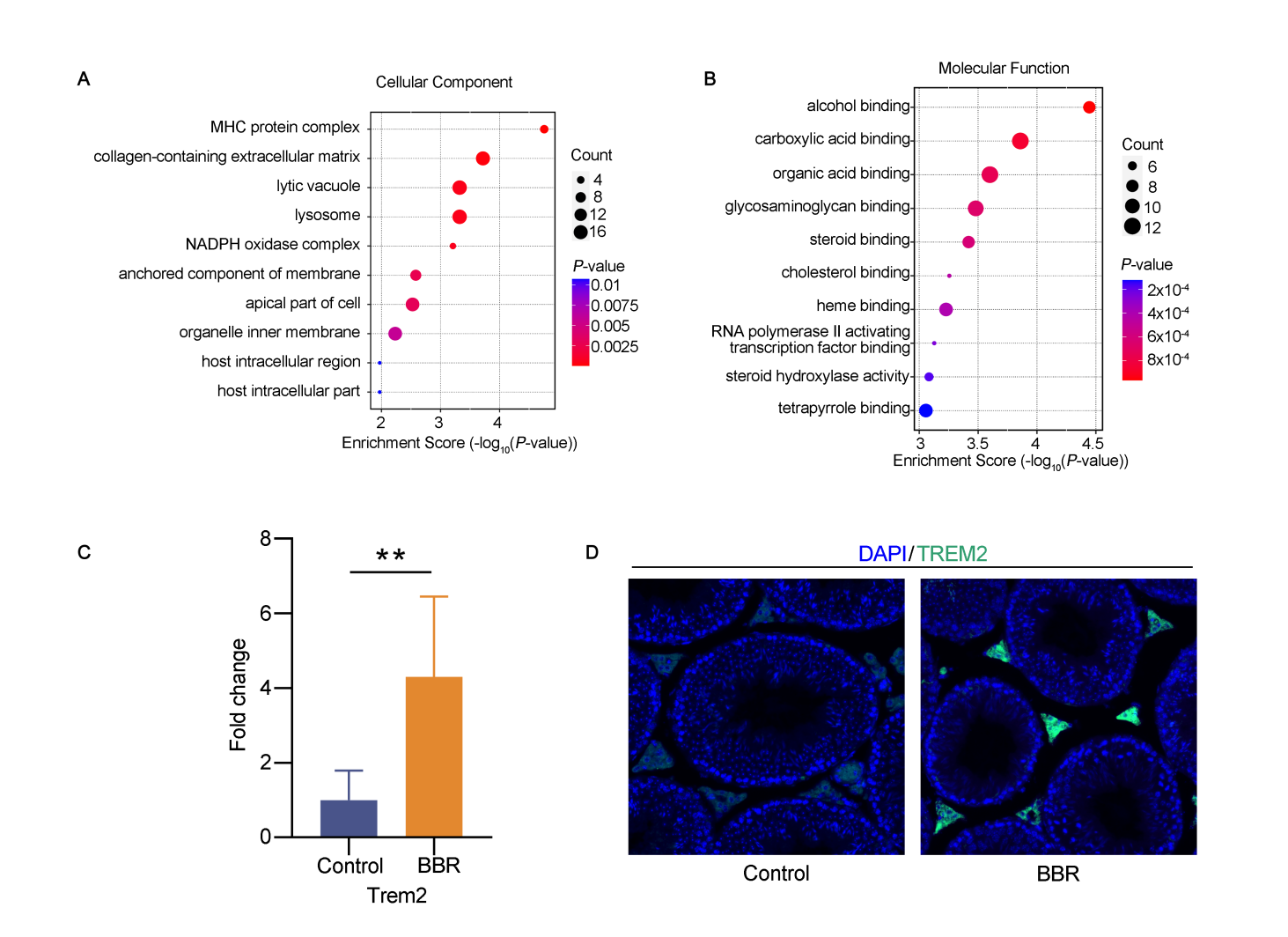
**

**Supplementary Figure S4. The functions involved in differentially expressed genes of testes from control and BBR-treated mice were analyzed and verified** (A) The cellular component involved in differentially expressed genes between untreated groups and BBR-treated groups was analyzed. (B) The molecular function involved in differentially expressed genes was analyzed. (C) *Trem2* mRNA levels in the testes from control and BBR-treated mice were analyzed by RNA sequencing. *n*=2 for each group. (D) Immunofluorescence analysis of the expression level of TREM2 in testis. Scale bar: 50 μm. Data are expressed as the mean ± SEM. Differences without statistical significance were not labeled; **P*<0.05, ***P*<0.01, ****P* <0.001.


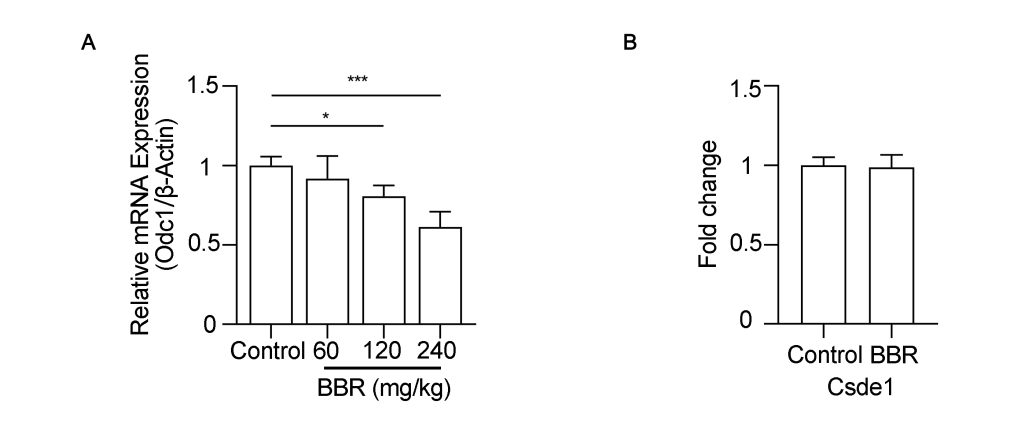


**Supplementary Figure S5. The decrease in mRNA level of *Ldlr* was not due to its degradation, but to a decline in ornithine** (A) *Odc1* mRNA levels in the testes from control and BBR-treated mice were analyzed by RT-qPCR. *n*=4 for each group. (B) *Csde1* mRNA levels in the testes from control and BBR-treated mice were analyzed by RNA sequencing. *n*=2 for each for each group. Data are expressed as the mean ± SEM. Differences without statistical significance were not labeled; **P*<0.05, ***P*< 0.01, ****P*<0.001.
